# 

**Published:** 1913-03-29

**Authors:** 


### Buildings Contemplated.

Ballymena.—Application for sanction to a loan of £7,500 is being made by the Guardians for the erection of a new hospital.

Colinton.—Plans have been approved by Edinburgh Town Council for new buildings at Colinton Mains Hospital for tuberculous patients. The buildings will be of timber construction on concrete foundations, and are to cost close on £3,C00.

Dundee.—Tenders are invited for alterations at West Green Asylum for Dundee District Lunacy Board. Particulars may be had from Mr. T. Martin Cappon, F.R.I.B.A., 32 Bank Street, Dundee.

Hambledon (Surrey).—Mr. E. L. Lunn, 36 High Street, Guildford, is architect for a nurses' home at the Workhouse Infirmary. Particulars for tenders may be had from the architect on deposit of £2 2s.

Haslingden.—Mr. H. Ross, A.R.I.B.A., 15 Cannon Street, Accrington, is architect for sundry works at the Workhouse premises. Tenders are invited.

Hull.—The City Architect, Mr. J. H. Hirst, has prepared particulars for alterations, etc., at the City Asylum premises, Willerby. A tender amounting to £2,689 has been received.

### The Growth of Walthamstow Hospital.

The annual meeting of the Walthamstow General Hospital was held on Wednesday last week. During the past year the Council have had to deplore the loss by death of two of its oldest members, Mr. George Baker and Mr. Benjamin Biggs, both of whom joined the council in 1894, the year when the Children's Cottage Home of six beds became a general hospital. The work of the hospital during the past year has so increased, particularly in surgery, that it had been found necessary to alter the duties of the house surgeon, and three honorary anæsthetists had been appointed and one honorary radiographer and pathologist. The number of in-patients treated during the year has been 597, and the number of out-patients has risen by nearly 1,500. The hospital has received grants from the King Edward Hospital Fund for London, £350 (£50 held over from their grant for 1911 for specified improvements, viz., new operating table and steriliser, which improvements have now been carried out). The average, however, of cost per patient per week is given as 16s. 10d. per head. In all the finances show a deficit of £369 16s. 10d., but the President (Sir T. Courtenay Warner, C.B., M.P.), upon hearing of the debt, sent a donation of £105, while others also have been received. Mr. Gilbert Houghton, who was re-elected treasurer, said that they had had 3,692 out-patients in 1910, 4,520 in 1911, and 5,877 in 1912, making an average cost of 1s. 2d. per patient. Some people thought that the Insurance Act put that right, but the Act had made practically no difference to them, and if it did so it would be on the wrong side. In Walthamstow they had a large number of uninsured persons to deal with. The Walthamstow Hospital, as previously mentioned, was opened in 1878 as a cottage home, and in 1894 as a general hospital with nineteen beds. An extension was opened in 1897 providing for thirty-five beds, and in 1903 further extensions were undertaken, which now give the hospital forty-five beds, with a thoroughly equipped out-patients' department, operating theatre, casualty room, and mortuary.

### Obituary.

Dr. Arthur John Wallace, M.D., of Liverpool, has died at the age of forty-six. Educated at Liverpool and Edinburgh, where he graduated in 1889, one of his first residential posts was that at the Liverpool Royal Infirmary. He was later appointed to the Holt Scholarship in Anatomy and Surgery at University College, Liverpool. Paris and Berlin were then visited for purposes of gynæcological study, which bore practical fruit in his subsequent appointment as assistant lecturer in gynæcology at University College. He became increasingly preoccupied with this branch of medicine, and had staff posts for many years at the Hospital for Women and at the Maternity Institution, Brownlow Hill. His most recent appointment was that of honorary consulting gynæcologist to the Royal Southern Hospital, while his reputation in this speciality was well acknowledged by his election in 1908 to the presidency of the North of England Obstetrical and Gynæcological Society.

### LEGACIES TO GLASGOW HOSPITALS.

Under the will of Miss Margaret Dolores Dunlop several Glasgow institutions receive handsome legacies. The Western Infirmary and the Royal Hospital for Sick Children get £2,000 apiece; the Broadstone Jubilee Hospital, £1,200; the District Nursing Association, Port Glasgow, £500; the Higginbotham Sick Poor Nursing Association, £500; and the Lockerbie District Nursing Association, £300.

### Future of Mount Vernon Hospital.

At a meeting of the London County Council last week Major Cecil Levita, the chairman of the Public Health Committee, informed Mr. J. A. Dawes, M.P., chairman of the London Insurance Committee, that, with respect to a scheme for dealing with tuberculosis in London, the possibility of acquiring Mount Vernon Hospital and the Stepney Sick Asylum would be considered. Mr. Dawes remarked that while the general scheme was being prepared the opportunity of acquiring the Mount Vernon Hospital and the Stepney Sick Asylum might be lost.

### Appointments.

Mr. Alfred Carleton Williams and Miss Helen Beatrice Hanson have been appointed medical assistants in the Public Health Department of the London County Council at a commencing salary of £400 a year.

The following appointments have been made at the Royal Free Hospital, Gray's Inn Road : Senior resident medical officer, Mr. C. A. Joll, F.R.C.S.; senior obstetric assistant, Miss Rawlins, M.B., B.S.; clinical assistant to Dr. Phear, Mrs. Westlake, M.B., B.S.

Dr. F. J. Wethered, F.R.C.P., has been elected to the staff of the Middlesex Hospital as fourth physician, on the retirement of Sir J. Kingston Fowler; and Dr. E. A. Cockayne, M.R.C.P., has been appointed a physician to the out-patients' department, in succession to Dr. Wethered.

The following appointments have been made in the Public Health Department of King's College : Colonel W. G. King, C.I.E., M.D., formerly Commissioner in the Madras Presidency, has been elected lecturer on applied hygiene; Dr. E. W. Routly, medical officer of health at Aldershot, lecturer in sanitary law; and Dr. W. F. Roach, lecturer on school hygiene for medical officers. It will not be forgotten that the public health laboratories of King's College and King's College Hospital are now conducted in a building in Chandos Street, formerly a part of the Charing Cross Medical School.

### Personalia.

Mr. Francis J. H. Wood and Mr. Ray Mond, respectively chairman and member of the committee of the Worcester General Infirmary, have resigned their posts.

The year 1912 has deprived the General Hospital, Birmingham, of many interesting workers in the hospital field, and the latest report draws attention to the loss by death of Mr. Alderman Charles Gabriel Beale, a vice-president, who was chairman of the orchestral and festival committees for many years, committees which mark an epoch in the history of hospital support in the city, and of Mr. Henry Lea, the consulting engineer to the hospital and for thirteen years a member of the board. Another resignation was that of Dr. D. C. Lloyd-Owen, ophthalmic surgeon. A portrait of Sir Thomas Chavasse, as The Hospital noted at the time, was presented to the hospital shortly before his death on February 17.

Mr. H. J. Godwin, F.R.C.S., surgeon to the Royal Hampshire County Hospital, Winchester, has received a very handsome testimonial from the members of the Winchester Division of the British Medical Association. Mr. Godwin has acted as hon. secretary of the division for seven years. The Chairman, Dr. H. Maturin, of Winchfield, alluded to the skill, tact, judgment, and common sense he had displayed during the Insurance controversy. The testimonial, as described in the *Hampshire Chronicle*, consisted of a silver tea kettle, a list of subscribers, and a silver tea tray, with gadroon and shell bordering, and the following engraved upon it : "Presented to Dr. H. J. Godwin by the members of the Winchester Division of the British Medical Association and other friends as a token of the very valuable services rendered by him on behalf of the profession during the passing of the National Insurance Act."

### Gifts and Bequests.

The National Hospital for Consumption, Ventnor, has received £100 from "A Friend, per J. D. L."

A special grant of £100 has been made by the Goldsmiths' Company to the Convalescent Home for Poor Children, St. Leonards-on-Sea.

The late Viscount Tredegar has left £5,000 to the Newport and Monmouthshire Hospital, £5,000 to the Cardiff Hospital, and £1,000 to the Brecon Hospital.

Mrs. Mary Constance Snape, widow of Dr. Ernest Alfred Snape, has left the residue of her estate in equal shares to the Hospital for Sick Children, Great Ormond Street, and four other institutions.

Mr. Edwin Tate has sent £500 towards increasing the accommodation of the Homœopathic Convalescent Home, Eastbourne, so that male patients as well as women and children may be received.

Among the smaller gifts by Mr. Alfred Jones's will to institutions are £3,000 each to the Liverpool Hospital for Women, the Home for Epileptics at Maghull, and the Liverpool Seamen's Orphan Institution.

